# Effect of Regret Aversion and Information Cascade on Investment Decisions in the Real Estate Sector: The Mediating Role of Risk Perception and the Moderating Effect of Financial Literacy

**DOI:** 10.3389/fpsyg.2021.736753

**Published:** 2021-10-29

**Authors:** Kaixin Wangzhou, Mahnoor Khan, Sajjad Hussain, Muhammad Ishfaq, Rabia Farooqi

**Affiliations:** ^1^School of Management, Hainan Medical University, Haikou, China; ^2^Institute of Business Management Sciences, Agriculture University, Faisalabad, Pakistan; ^3^Faculty of Management Sciences, Foundation University, Islamabad, Pakistan; ^4^Faculty of Management Sciences, Riphah International University, Faisalabad, Pakistan; ^5^Department of Psychology, University of Central Punjab, Lahore, Pakistan

**Keywords:** regret aversion, information cascade, financial literacy, investment decision making, risk perception, real estate

## Abstract

The real estate sector plays a significant role in the economy of any country. However, many investors make irrational investments in the real estate market. Therefore, the purpose of this study is to assess the effects of regret aversion and information cascade on investment decisions while considering the moderating role of financial literacy and the mediating effect of risk perception in the real estate sector of developing countries. This research utilized a quantitative research technique, collecting data by distributing structured questionnaires to real estate investors, followed by convenience sampling. This study used both descriptive and inferential statistics to make the data more meaningful. SPSS 25.0 was utilized to interpret the data. Cronbach's alpha was used to test for internal consistency, while validity was checked through correlation. Confirmatory factor analysis (CFA) was applied to confirm that the items on the questionnaire are perfectly loaded on their construct. Furthermore, process macro, model 5, was used to investigate the moderation mediation. This work addresses a gap in the literature by studying financial literacy as a moderator and risk perception as a mediating variable in regret aversion bias and information cascade bias's relationships with investment decisions in the real estate sector. The results confirmed that financial literacy weakens the negative effect of behavioral biases (regret aversion and information cascade) on investment decisions. In addition, risk perception mediates the relationships between these cognitive biases (regret aversion and information cascade) and decision making. The effects of other behavioral biases in real estate and stock market contexts should be examined in future research.

## Introduction

Behavioral finance assumes that imperfect information leads to irrational investment decisions. Behavioral psychology has provided new insights into traditional psychology by introducing new finance concepts such as financial knowledge, cognitive biases, and risk perception (Bazley et al., 2021). Behavioral finance paradigms highlight that investment decisions of people are not based solely on market information, as thoughts, emotions, and judgment errors of the individuals are also reflected in these decisions. The study of behavioral finance originated from the ideas put forth by Tversky and Kahneman and their prospect theory, which follows from the expected utility theory. Prospect theory suggests that investors are more likely to focus on gains rather than the perceived risk of loss when the outcome of an investment is uncertain. On the other hand, regret theory relies on two basic assumptions: first, that many individuals experience feelings called regret and joy and, second, that people consider these feelings when making decisions in uncertain situations (Rasheed et al., 2018; Kaur and Bharucha, 2021; Zhuo et al., 2021).

Biases can be thought of as representations of an investor's mind. Cochrane reported that biases are not correlated with the correctness of a decision based on its outcomes. Furthermore, biases can arise in different ways, often due to irrational or overly positive attitudes. Behavioral biases provide the fundamental reasoning behind irrational investment decisions (Ahmad, 2020). Investors can minimize risk only if they make their choices based on rational and irrational decisions (Naseem et al., 2021). When deviations occur systematically from norms and thinking, cognitive biases arise (Weber, 2004; Djalilov and Ülkü, 2021).

Savage (1951) reported that investors cannot bear losses because it confirms that their initial judgment was wrong. Regret aversion encourages others to admit their mistakes to avoid future regret (Thaler, 1985). Regret aversion is a situation in which individuals forgo making a potentially incorrect investment decision to ward off the unpleasant feelings that could arise (Frehen et al., 2008). Regret aversion is a concept within the prospect theory (Kahneman and Tversky, 1979) describing a negative emotional bias that urges investors to avoid regret, thus sometimes making the wrong decision. Tsiros and Mittal (2000) also investigated that regret aversion is a significant negative emotion. Zeelenberg et al. (1996) argued that regret theory is action-based. Many studies support the general observation that decision makers are averse to regret and try to avoid regretting decisions.

The term “information cascade” refers to a circumstance in which it is optimal for a person to follow specific steps—to follow the behavior of someone else—without taking into account that person's information (Bikhchandani et al., 1992). According to Spoerri (2008), an information cascade involves agreeing with the opinions of others despite having incomplete information—however, some of the missing information must be included if a rational decision is to be made. Information cascades reduce decision independence, thereby weakening the real estate market (Brzezicka et al., 2018). Information cascades prevent decision makers from sufficiently understanding the actual value of products. Thus, they form their opinions of the value of products by observing their predecessors and society. The influence of behaviors of other individuals can be so great that it dominates their information from decision makers (Alevy et al., 2007). Therefore, under these circumstances, predecessors are imitated by the decision makers, regardless of how much and what kind of information they receive (Duan et al., 2009). Individuals ignore their private information when making decisions, and herd behavior occur as a substantial number of individuals make the same decision while not necessarily ignoring their private information (Çelen and Kariv, 2004; Levy et al., 2020).

The term “risk perception” refers to how investors perceive the risk of financial assets based on their concerns and experiences (Ahmad and Shah, 2020). Moreover, the concepts of risk and uncertainty are correlated in determining the intensity of risk as perceived by investors (Ishfaq et al., 2017). Risk perception is a vital component of the decision-making process. Investors and policymakers try to minimize risk when investing or developing projects. Naturally, investors who have deep knowledge of the financial market perceive risk more accurately than inexperienced investors (Nguyen and Rozsa, 2019). Risk perception is connected to the beliefs, thoughts, and judgments of an investor, and all investors perceive risk differently, as various choices are influenced by the makeup of each investor's portfolio. Weber et al. (2002) expressed that risk perception influences the willingness of an investor to take risks.

Extending our knowledge of the mediating mechanisms within this link and building on prospect theory, we proposed that risk perception mediates the effect between cognitive biases and irrational decision making. Risk perception is related to the subjective judgment of the investors, and it deals with their perceptions and the severity of risk (Singh and Bhowal, 2010). Specifically, it reflects the thoughts of individuals when they are asked to evaluate uncertain or risky activities (Slovic, 1987; Sartori and Ceschi, 2011). Risk-taking is a common practice for investors when making decisions. Therefore, without considering the risk perceptions of investors, cognitive biases cannot fully describe the decision-making processes of investors, thoughts of investors, and the errors in the judgments of investors, which shape their perceptions (Pandey and Jessica, 2018).

Financial literacy is defined as the ability to make effective decisions about using and managing the money of an individual (Nguyen et al., 2016). Financial literacy also reflects the ability of people to understand and use financial information with confidence and skill (Huston, 2010). Ramalho and Forte (2019) examined that financial literacy is the main component of any successful strategy to find solutions for improving the lives of a local population. Financial literacy is usually seen as a specialized kind of investor knowledge related to how successfully a person manages their financial affairs (Alba and Hutchinson, 1987). According to Giesler and Veresiu (2014), an investor's ability to determine how money works and how investors use it to maximize profits when someone pledges to generate more money is due solely to that investor's financial literacy. People with financial literacy understand financial products and markets better than other people and are more likely to accurately assess their financial data resources (Lusardi and Mitchell, 2011). Financial well-being is positively linked to the individual-level quality of life and mental and physical health (Blanchflower and Oswald, 2004). Financial literacy also helps to strengthen interpersonal relationships and work performance (Brüggen et al., 2017).

Previous studies have been carried out to check the heuristic effect on the investment decisions by real estate investors (Gitau et al., 2019). This study will contribute to the literature on financial literacy as a moderator in the real estate sector and, thus, will be beneficial for practitioners and researchers by increasing their knowledge of real estate investments. This study is, therefore, set out to assess the influence of regret aversion and information cascade on real estate investment. Lones and Peter (2000) stated that the decision-making process related to real estate investments involves the systematic search, application, acquisition, and analysis of the required real estate corresponding to the economic and personal goals of investors during the period of ownership. According to Norizan and Pee (2021), real estate is the most important asset of any economy, and it should be investigated through the behavioral aspect of an investor.

The contribution of this study is its analysis of the mediating effect of risk perception in the relationship between cognitive biases and investment decisions. Building on prospect theory, we proposed that risk perception mediates the effects of cognitive biases on irrational decision making. This study makes another contribution to the literature by showing that financial literacy strongly affects the decisions of investors to invest in stocks. Therefore, financial literacy contributes to the body of knowledge as a moderating variable.

**Figure 1:**
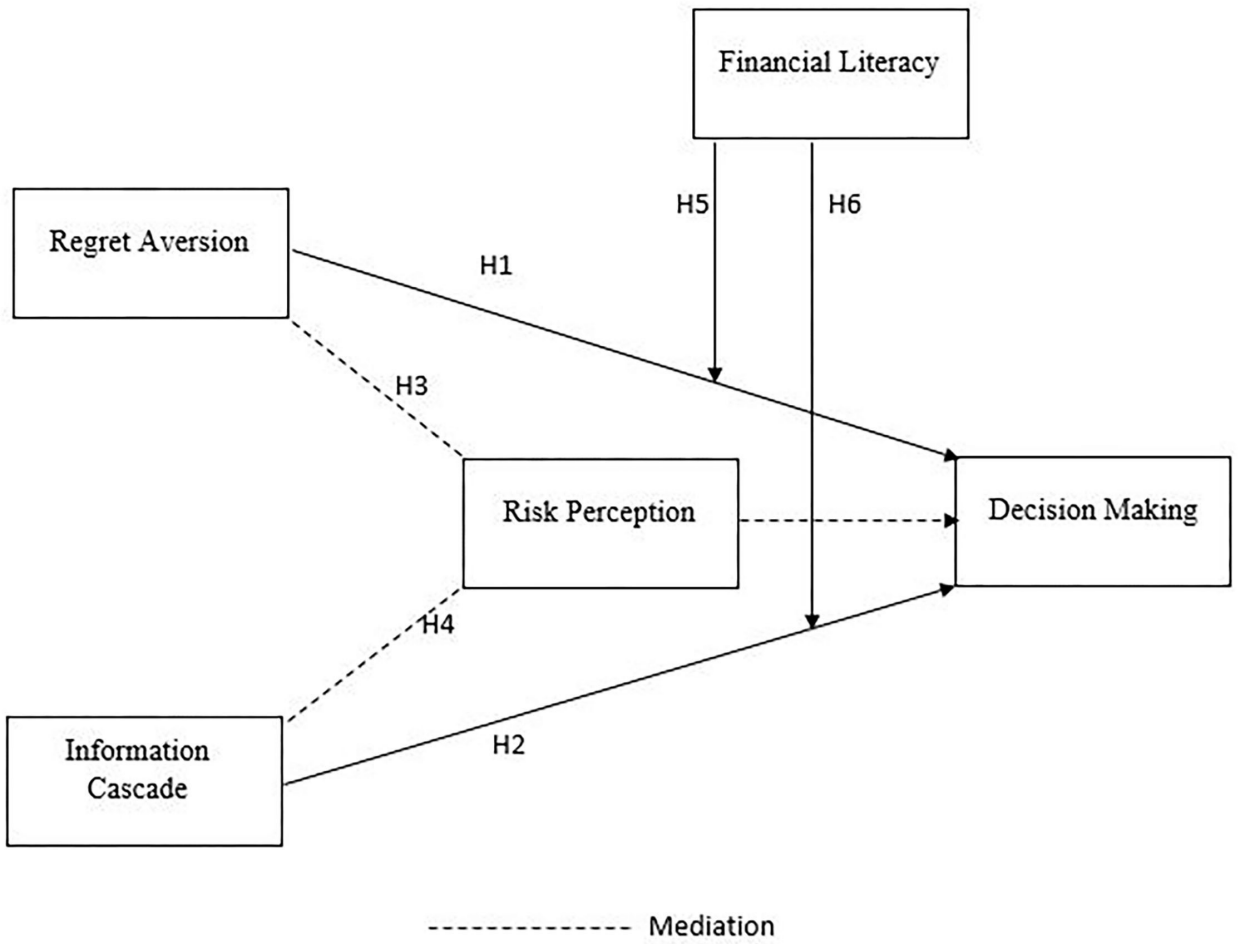
Conceptual framework.

The objective of this study is to determine how cognitive biases affect irrational decision making in the presence of risk perception and financial literacy (Figure 1). The results indicate that risk perceptions of investors underlie the relationship between cognitive bias and irrational decisions of investors, thus advancing the general understanding of how cognitive biases affect irrational decisions, both directly and indirectly, *via* risk perception.

## Theoretical Background

### Prospect Theory

Behavioral finance is a discipline that analyzed the behavioral influence of psychology on the decisions of investors across different sectors and industries. According to Zahera and Bansal (2018), the key educational and parental activities in the field of behavioral finance are two psychologists Kahneman and Tversky (1979), who first introduced the prospect theory. They explained that investor decisions are based on potential gains and losses rather than on final results. Moreover, Daniel and Tversky stated that prospect theory gave decision-making significance to a person. Prospect theory was popular as an alternative to the expected utility theory, effective market hypothesis, and rational expectation theory (Zahera and Bansal, 2018). As Thaler (1980) developed theories on applying prospect theory on financial markets and as a financial theorist, he argues that individuals do not always behave rationally but often make mistakes while making investment decisions.

### Regret Theory

Fishburn (1982), Loomes and Sugden (1982), and Bell (1982) devised the regret theory in 1982 and described the empirical and normative state of regret theory. Yang and Wang (2018) stated that regret theory could be used as an alternative behavior theory for apprehending regret aversion. The theory of regret is based on two basic assumptions: first, many individuals experience feelings called regret and joy; and second, when deciding on the situation of uncertainty, it wants to anticipate and take into account these feelings.

### Information Cascade Theory

Bikhchandani et al. (1992) proposed information cascade theory widely applied to explain the behavior of people in various fields, including information systems and finance. Wang et al. (2018) stated that according to information cascades theory when information about a person is limited, he can make decisions based on the behavior of others. In the past, herd behavior and information cascades were used interchangeably (Çelen and Kariv, 2004). Lones and Peter (2000) indicated a significant difference between information cascades and herding behavior. Herd behavior refers to a situation in which people imitate the actions of others, and thus to some extent, ignore their information and judgment about the merits of their decisions (Kahan and Klausner, 1996). While the information cascade creates a situation where decision makers only do not sufficiently understand the real value of the product so that they develop its usefulness from observing their predecessors and society (Duan et al., 2009).

## Hypotheses Development

### Regret Aversion and Decision Making

Loomes and Sugden (1982) came up with the first definition of regret aversion and explained that it motivates individuals to prevent future regret. According to Sattar et al. (2020), regret aversion means that the investor never wants to regret inefficient investment decisions. Chen et al. (2018) investigated that during decision making, the human response often encounters regret aversion preferences and decision makers feel regret if they make the wrong decision. Regret aversion is a consequence of expanding the theory of prospect (Kahneman and Tversky, 1979). In addition, investor emotions, feelings, and intuitions influence their decisions and lead to irrational behavior (Kahneman and Tversky, 1979). According to Zahera and Bansal (2018), when people regret a decision, this has a larger influence on their psychological effect. Either they take more risks, or they compel from risk; this is done to prevent the pain of regret in the future.

Regret aversion seems to be the primary emotion in making decisions (Loomes and Sugden, 1982). Moreover, regret aversion is a negative emotional bias in which the investor wants to avoid regret in the past and take the wrong decision. Shimanoff (1984) found that common negative emotion of an investor is regret aversion. Moreover, regret aversion bias causes to hold losing assets so long and avoid investing in lower assets at falling prices (Shiller, 2003). Isidore and Christie (2019) stated that regret aversion is a bias that postpones a decision that leads to regret. Zeelenberg and Pieters (2004) state that regret is directly connected with the choice or decision at hand. Many studies support the general observation that decision makers are averse to regret and try to ward off their decision making (Larrick and Boles, 1995; Zeelenberg, 1999; Mellers, 2000; Zeelenberg and Pieters, 2004). As per the above discussion, it can be hypothesized as:

*H1: There is a significant negative effect of regret aversion on investment decision making*.

### Information Cascades and Decision Making

The phenomenon of information cascades picked up conspicuousness in economic research in the 1990s (Bikhchandani et al., 1992). It is an activity that leads to another opinion on incomplete information that one has to make a rational decision. Decision making of investors is influenced by judgment errors, feelings, and thinking (Pfnur and Wagner, 2020). Moreover, Wang et al. (2018) stated that the information cascade is based on information cascade theory. People take their decisions by following the behavior of others when their information is limited. Information cascades are more common on the market in times of more significant uncertainty. Seiler (2012) argued that if it is true that uncertainty and information cascades have a significant correlation, real estate must be much more sensitive to information cascades than the stock market. In an economic and financial environment where decision makers have incomplete information about the real estate sector, it cannot be rational to ignore their personal information and make choices based on what others consider (Alevy et al., 2007; Kim and Lee, 2008).

Wang et al. (2018) investigated that herding behavior reveals itself as a reflection of an information cascade that makes the opinions of people more and more consistent. Huber et al. (2014) explained that information cascades are a form of rational herding. It is believed that the first decisions of others create an environment in which later decision makers rationally ignore their private information following others. It is observed that when decision makers do not know exactly the actual value of the product, so its usefulness is based on observing the behavior of its investors. The influence of the behavior of others can be so vital that it is the product that influences its information dominated by decision makers (Duan et al., 2009). As a result, although options are available as an immediate replacement, information cascades can lead to investment decisions dominating one another and sometimes to the rejection of a more efficient decision of investment (Abrahamson, 1991). As per the above discussion, it can be hypothesized as:

*H2: There is a significant negative effect of the information cascade on investment decision making*.

### Meditating Role of Risk Perception

The term “risk perception” indicates how investors examined financial assets risk based on their experiences and concerns (Ahmad and Shah, 2020). Knight (1921), an economist, has first defined the concept of uncertainty and risk, distinguishing between measurable or non-measurable. According to Daskalaki et al. (2014), there will be no risk without uncertainty. Risk perception is correlated with risky decisions. In addition, risk perception is a vital determinant in forming risky decisions (Sitkin and Weingart, 1995).

According to Houghton et al. (2000), behavioral biases directly influence risk perception. Investors often become emotional and create bias in their investment decisions, leading to irrational decision making.

Risk perception plays a fundamental role in the behavior of investors (Alam et al., 2020). Investment, as an expense for future benefits and profits, is tangled with risk and uncertainty. In addition, Jackson and Orr (2019) investigated that uncertainty in the investment decision-making process could result in asset prices deviating from their market value and that risky behavior may seem irrational. Risk perception is related to the subjective judgment of intensity and severity of risk (Slovic, 1987). According to Kahneman and Tversky (1979), the trade-off of investors exists between profit margins and the level of risk when deciding.

Moreover, Sindhu et al. (2014) examined the cause-and-effect relationship between risk perception and investment decision; their results showed that risk perception and past profits heavily influence the decisions of investors. According to Daskalaki and Skiadopoulos (2016), risk perception determines the investor's view of when to assess the past or how the risk is related to the investment decisions. Moreover, Robinson and Marino (2013) suggested that the risk perception of investors is negatively related to investment decisions.

In previous researches, risk perception is used as an intervening variable. Moreover, Ishfaq et al. (2020) examined that the analysis of meditation determines the mediation process leading from the transmitted variable toward the criterion variable (decision making). Simon also investigated that risk perception mediated the relationship between cognitive biases and the decision to start a venture. They also proposed that other biases need to be considered for future research. Nguyen and Rozsa (2019) investigated that risk perception and risk tolerance significantly affect investment decision making.

An individual's behavioral biases directly affect risk perception and investment decisions (Ishfaq et al., 2017). In addition, behavioral biases indirectly influenced investment choices through their impact on risk perception, which means that risk perception plays a mediating relationship (Ishfaq et al., 2020). The mediation analysis process assumes that the third variable (risk perception) shows a generative mechanism through which independent variables (regret aversion and information cascade) influence a dependent variable (investment decision). As per the above discussion, it can be hypothesized as:

*H3: Risk perception mediates the relationship between regret aversion and investment decisions*.
*H4: Risk perception mediates the relationship between information cascade and investment decisions*.

### The Moderating Role of Financial Literacy

Noctor et al. (1992) consider financial literacy as the ability to make informed decisions and make effective decisions about using and managing money. Bellofatto et al. (2018) examined financial literacy as the ability to process financial information and make informed decisions about financial planning, pensions, wealth accumulation, and debt (Lusardi and Mitchell, 2014).

Financial literacy is the essential information individuals ought to survive in modern society (Kim, 2001). Underlying financial literacy is less than desirable, so lack of financial literacy has become a worldwide issue (Lusardi and Mitchell, 2011; Sahin et al., 2016). According to Agnew and Szykman (2005), people in the US have greater financial education than in developing countries. According to Jiang et al. (2019), investors with higher education and more experience have greater financial literacy. Moreover, Jariwala (2015) found that financial literacy has an essential effect on the investment decisions of investors. In addition, financial literacy is closely related to the financial prosperity of an individual, with a positive relationship between higher levels of financial literacy and better investment decisions (Khan et al., 2018). According to Nguyen and Rozsa (2019), financial literacy plays a vital role in improving investment decision making and is also for sound financial decisions.

Behavior biases and financial literacy is essential to investigate for understanding the actual behavior of investors. Behavioral biases were examined in the developed countries by many researchers, and they all argued that decisions of most investors include biases that affect their investment decisions (Kahneman and Tversky, 1979; Odean, 1998a,b; Weber and Camerer, 1998). Idris et al. (2017) investigated financial literacy levels and investment decisions relationship and found that highly educated investors supported the use of different investment decision-making techniques than low literate investors.

Many studies showed that both financial education and biases influence investor behavior (Robinson and Marino, 2013). Regret aversion is a bias that affects investment decisions, and if investors are financially literate, it will change its effect. Moreover, much of the literature shows that literate financial investors behave more rationally in financial matters (Hilgert and Hogarth, 2002).

*H5: Financial literacy moderates the relationship between regret aversion and investment decision making*.

Financial literacy plays a significant role in investment decisions when investors use financial information as the basis for their saving, investment, and borrowing behavior. Financial literacy is an essential factor that describes why investment decisions differ from many investors (Idris et al., 2013). Financial literacy is essential in investment decisions, and informational cascades negatively affect investment decision making, so a financially literate person can change its effect. As per the above discussion, it can be hypothesized as:

*H6: Financial literacy moderates the relationship between information cascade and investment decision making*.

## Methods

### Target Population and Sample Size

Statman (2014) states that ordinary people behave irrationally and make mistakes due to behavioral effects. The sample represents an element of data collection and a fragment or part of a population selected for a survey. According to Kline (1998) and Hair et al. (1998), an acceptable sample size yields a ratio between 5 and 10 observations per estimated construct. Therefore, the sample size is developed by multiplying the total number of items by 10. Thus, as this research used 20 items, the sample size is 200.

### Data Collection

Research questionnaires were distributed to real estate investors. A pilot study was conducted before the questionnaires were distributed to the respondents. The investors in the real estate business are uneducated, so the items were converted into the Urdu language. A sample questionnaire was validated (face and content validity) by two Ph.D. faculty members of GC University and a group of real estate investors with MBA degrees. Item 4 of financial literacy and the second item of regret aversion were edited (Urdu language) so that respondents could better understand the items and eliminate common method bias. Regarding the general description of the sample, data were collected using a structured questionnaire and sent to 325 real estate respondents—that is, 325 questionnaires were distributed to different retail investors. Out of these 325 questionnaires, 287 fully completed questionnaires were received from respondents. Another 17 returned questionnaires were discarded due to incomplete information. The remaining 21 questionnaires were not received from respondents. Overall, the response rate was 88% (Table 1).

**Table 1:** Breakdown of sample size.

**Particulars**	**No. of questionnaires distributed**	**Percentage (%)**
**Composition of Questionnaire**		
Questionnaires distributed	325	100
Questionnaire completed	287	88.3
Questionnaire discarded	17	5.23
Questionnaire not received	21	6.46

The convenience sampling method (also known as availability sampling) was used to select the respondents, as this method provides the highest response level while saving resources and time (Bryman and Bell, 2011). Convenience sampling is a specific type of random sampling method that depends on collecting data from members of a population who are readily available to participate in the study. According to Bornstein et al. (2017), convenience sampling is a time-saving way to collect data quickly from a large group of people.

### Measurement

Questionnaires were used to measure the study constructs and collect data from the population. Time lagged consists of 2 months apart, and data were collected from 287 respondents. The items on the questionnaire were answered on a five-point Likert scale, with possible responses ranging from “strongly disagree” to “strongly agree.” Regret aversion was measured using the five items proposed by Thaler (1985). A sample item is “After selling the profitable properties, I will be upset with losing properties that have not been sold yet.” The risk perception scale was measured using the four items proposed by Weber et al. (2002). A sample item is “I Invest 10% of my annual income in a moderate growth mutual fund.” Information cascade was measured by the three items proposed by Bikhchandani et al. (1992). A sample item is “I use my information to make investment decisions.” Financial literacy was measured by the three items proposed by Nguyen et al. (2016). A sample item is “Self-rating of overall knowledge of financial matters.” Decision-making was measured using the five items proposed by Scott and Bruce (1995). A sample item is “When making an investment, I trust my inner feelings and reactions.”

### Demographic Variables

This study analyzed the characteristics of respondents pertaining to demographic variables and real estate investment decisions. In the present research, demographic variables consist of male and female respondents. Age was divided into five different age groups: 20–25, 26–30, 31–35, 36–40, and above 41. The monthly incomes of respondents also consisted of five different groups: 20,000 to 25,000; 25,001 to 30,000; 30,001 to 35,000; 35,001 to 40,000; and above 40,000 (Table 2).

**Table 2:** Demographic profile.

**Variables**	**Frequency**	**Percentage**
**Age (in years)**		
20–25	16	5.57
26–30	43	14.98
31–35	59	20.55
36–40	86	29.96
Above 40	83	28.91
**Gender**		
Male	232	80.83
Female	55	19.16
**Income**		
20,000–25,000	13	4.52
25,001–30,000	70	24.39
30,001–35,000	87	30.31
35,001–40,000 and above	117	40.76

### Statistical Analysis

All valid data obtained from respondents through the survey are analyzed by descriptive and inferential statistics. All questionnaires were carefully examined for incomplete and inappropriate answers. Final questionnaires are included in the SPSS 25. Descriptive analysis was performed on SPSS 25. In this section, the statistical analysis consists of the frequency distribution of gender, age, monthly income of the respondent, and experience of the respondent in real estate measured based on descriptive statistics. The normal distribution is also applied to check the skewness and kurtosis of the respondent's results.

Furthermore, inferential statistics consisted of correlation analysis and correlation coefficient used to test or check the relationship of study variables—reliability used in this research to check the internal consistency of the variable. Pearson's correlation was utilized to measure the validity of the constructs. Hayes (2013) process macro is utilized to test the moderation of financial literacy among regret aversion bias and information cascade bias and decision of investment and check the direct and indirect effects of variables on investment decision that is the dependent variable.

### Reliability and Validity

Results are reliable when the same results arise after applying statistical techniques. The reliability of data is investigated using the measure of Cronbach's alpha (**Table 5**). The validity of the measurement determines whether a tool can provide a measurement of a concept (Bryman and Bell, 2011). The correlation coefficient in the present case is statistically significant at α = 0.05, so the questionnaire can be considered consistent and valid for measuring what it was designed for. Pearson's correlation was used to test validity in the current research. Pearson's correlation was utilized to explain the relationship between two or more variables, as well as to indicate the direction and strength of these relationships. Data were analyzed using structural equation modeling (SEM) and confirmatory factor analysis (CFA) in AMOS 25.0.

## Results

### Descriptive Statistics

Fifty-five percent of respondents had a master's degree, and 45 percent had an undergraduate degree. The average age and experience of the respondents were 45.61 and 8.45 years, respectively. A time-lagged design was used to reduce common method variance (Podsakoff et al., 2003). Therefore, Herman's single factor was also calculated to examine the data for common method variance (Hair et al., 2010).

Demographic variables are controlled variables like gender, age, and income. These factors affect investment decisions. For example, Hassan Al-Tamimi and Anood Bin Kalli (2009) reported that demographic characteristics influence decision making and financial literacy. However, in the study, demographic variables had an insignificant effect on decision making (Table 3).

**Table 3:** Correlations.

	**Age**	**Income**	**Gender**	**Regret**	**Infor**	**Risk**	**Literacy**	**Decision**
Age	1							
Income	0.052	1						
Gender	0.021	0.101	1					
Regret	0.001	0.171^**^	0.023	1				
Infor	0.038	0.092	0.054	0.340^**^	1			
Risk	0.014	0.080	0.153^**^	0.124^*^	0.103	1		
Literacy	0.043	0.054	0.032	0.286^**^	0.431^**^	0.005	1	
Decision	0.074	0.010	0.001	0.042	0.059	0.115	0.151^*^	1

** *P < 0.001,*

* *P < 0.01*.

As shown in Table 4, the feedback of respondents aligns with the recommended ranges of skewness and kurtosis of +3 to −3. George (2011) reported that the skewness and kurtosis values must be in between +2 and −2. Moreover, Ghasemi and Saleh (2012) also noted that in large samples (200 or more participants) with small standard errors, the ranges for skewness and kurtosis should be changed to + (–) 2.58 instead of + (–) 1.96.

**Table 4:** Skewness, kurtosis, and factor loading.

**Sr. #**	**Item**	**Description**	**Skw**.	**Kurt**.	**FL**
1	RA1	After selling the profitable properties, I will be upset with those loosing properties once that have not been sold yet.	−0.292	−0.641	0.680
2	RA2	I will feel regret and disappointed if the price of the property, I sold keeps growing.	−0.131	−0.824	0.710
3	RA3	I don't want to buy those properties that are obviously overvalued.	0.131	−1.035	0.831
4	RA4	I sell profitable properties because I am afraid that, the property price would fall again.	0.023	−0.926	0.814
5	RA5	I pay additional cash for getting the extra benefit from property.	0.091	−1.021	0.833
1	IC1	I use my own information to make investment decisions	−0.222	−0.979	0.761
2	IC2	I think people's information are important than the information I have.	−0.244	−0.855	0.834
3	IC3	I always change my investment decision as per people's information.	−0.424	−0.748	0.621
1	FL1	Self-rate of overall knowledge of financial matters.	−0.226	−0.921	0.768
2	FL2	I am knowledgeable about investing.	−0.014	−1.06	0.872
3	FL3	I am confident about my ability to invest.	−0.209	−0.726	0.864
1	DM1	When making an investment, I trust my inner feelings and reactions.	−0.490	−0.69	0.875
2	DM2	I generally make investments that feel right to me	−0.340	−0.728	0.944
3	DM4	When I make an investment, it is more important for me to feel the investment is right than have a rational reason for it	−0.224	−0.875	0.836
4	DM5	When I make Investment, I tend to rely on my intuition	−0.510	−0.595	0.758
1	RP1	I Invest 10% of my annual income in a moderate growth mutual fund.	−0.326	−1.02	0.532
2	RP2	I invest 5% of my annual income in a conservative stock	−0.316	−1.06	0.943
3	RP3	I invest 10% of my annual income in government bonds (treasury bills).	−0.240	−1.11	0.778
4	RP4	I invest 5% of my annual income in a very speculative share.	−0.241	−1.31	0.889

*RA, regret aversion; IC, information cascade; FL, financial literacy; DM, decision making; RP, risk perception*.

### Reliability Statistics

According to Ali et al. (2019), the reliability of data was verified by the value of Cronbach's alpha. Cronbach's alpha value establishes the construct reliability (Pandey and Jessica, 2019). If Cronbach's alpha is higher than 0.7, it indicates a good fit (Fornell and Larcker, 1981). According to Hair et al. (2006), in reliability tests of internal consistency, Cronbach's alpha values above 0.7 are desirable. In general, a coefficient ≥0.7 is considered acceptable and is a good indicator of construct reliability (Nunnally, 1978). The value of Cronbach's alpha against regret aversion is 0.796 (Table 5). Moreover, information cascade is the independent variable and its Cronbach's alpha is 0.912. The values of Cronbach's alpha against risk perception, financial literacy, and decision making are 0.817, 0.848, and 0.846, respectively.

**Table 5:** Reliability statistics.

**Variables**	**Cronbach's alpha**
Regret aversion	0.796
Information cascade	0.912
Risk perception	0.817
Financial literacy	0.848
Decision making	0.846

### Moderation and Mediation Results

In this study, direct, indirect, and moderation effects are hypothesized and tested. The results (β = −0.270) reported in Table 6 show significant negative effects of regret aversion and risk perception. The results also reveal that the direct and indirect effects in the relationship between the variables and the output in terms of partial mediation are significantly negative.

**Table 6:** Direct and indirect effects and 95% confidence intervals.

	**β**	**Lower limit**	**Upper limit**
Information cascade → risk perception	0.20^*^	0.11	0.47
Information cascade → decision making	0.19	0.01	0.48
Risk perception → decision making	−0.24^*^	0.07	0.40
Standardized indirect effects			
Information cascade → risk perception → decision making	0.05^*^	0.12	0.16

* *Empirical 95% confidence interval does not overlap with zero. n = 287 (a sample of size 2,000 for bootstrapping)*.

**Figure 2:**
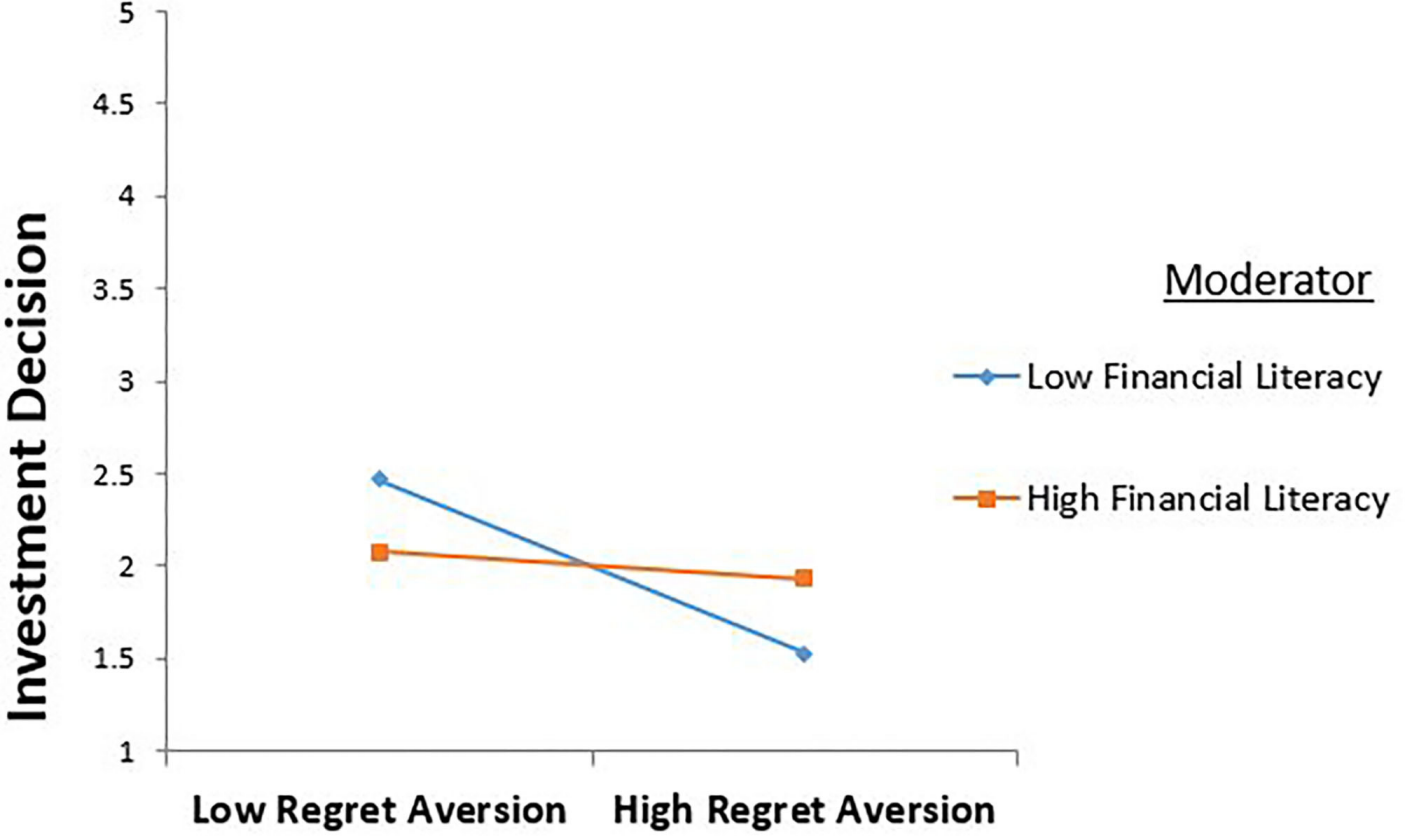
Moderating role of financial literacy between regret aversion and investment decision.

**Figure 3:**
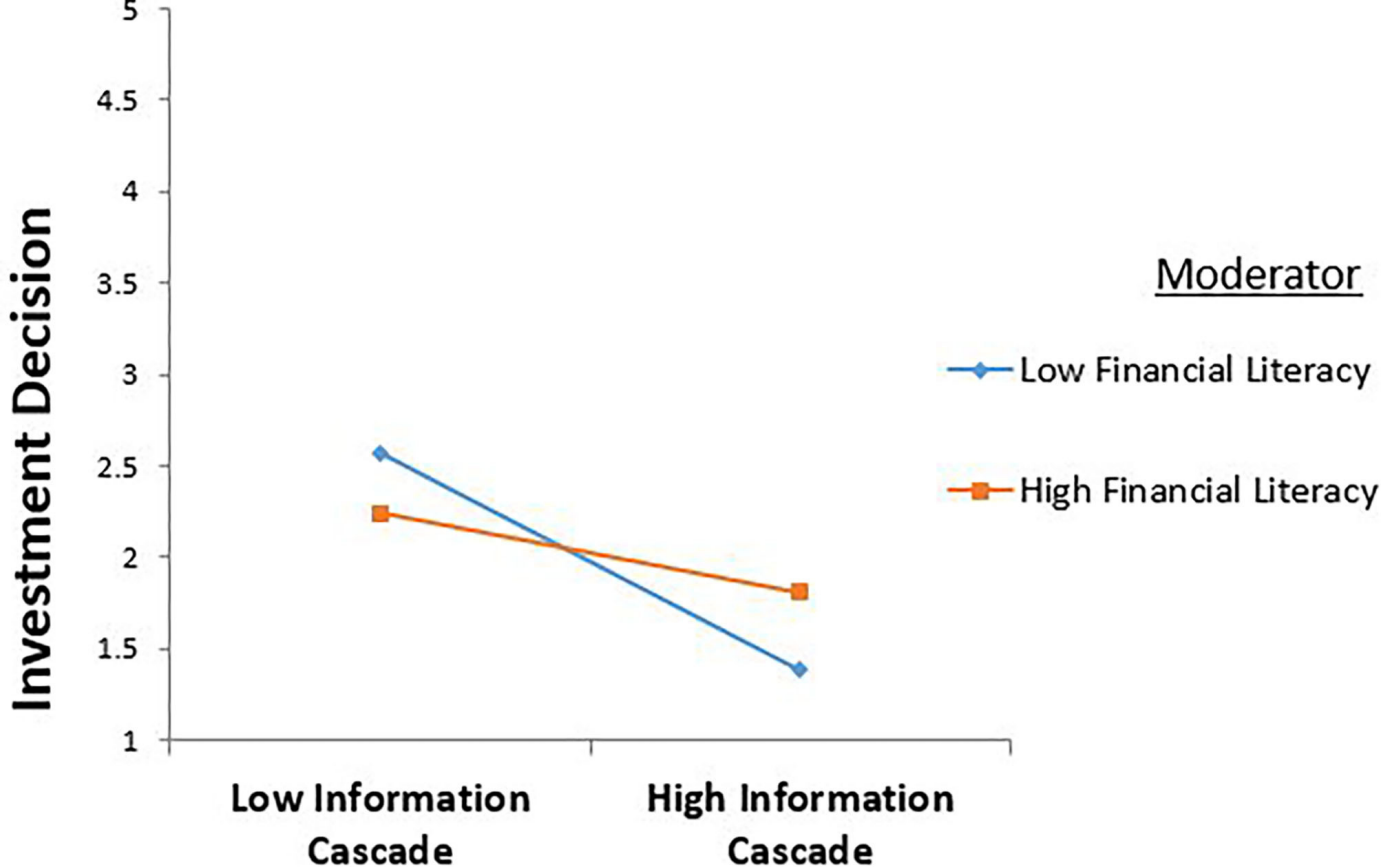
Moderating role of financial literacy between information cascade and investment decision.

The results in Table 7 and Figures 2, 3 show a significant negative effect of information cascade on risk perception and decision making. A partial mediation was also found between the predictor, mediating, and the outcome variables, as all effects are significant. Hayes (2013) also reported that the results must be significant in all directions to satisfy the condition of partial mediation.

**Table 7:** Direct and indirect effects and 95% confidence intervals.

	**β**	**Lower limit**	**Upper limit**
Regret aversion → risk perception	−0.30^*^	0.10	0.47
Regret aversion → decision making	−0.18	−0.03	−0.38
Risk perception → decision making	−0.24^*^	0.07	0.40
Standardized indirect effects			
Regret averson → risk perception → decision making	−0.07^*^	0.02	0.17

* *Empirical 95% confidence interval does not overlap with zero. n = 287 (a sample of size 2,000 for bootstrapping)*.

The results show that the composite reliability value is >0.8. The results are in line with Hair et al.'s (1998) recommendation that CR values should be >0.8. Moreover (Table 8), the value of AVE is >0.5, and square root of AVE is greater than its paired correlation. Convergent validity explains the extent to which all items are perfectly loaded on any given construct. A factor loading value of >0.5 suggests that all items are perfectly loaded.

**Table 8:** Discriminant validity.

	**CR**	**AVE**	**MSV**	**RA**	**IC**	**RP**	**FL**	**DM**
RA	0.912	0.776	0.604	**0.881**				
IC	0.897	0.637	0.224	0.07	**0.798**			
RP	0.87	0.532	0.438	0.064	0.474	**0.729**		
FL	0.925	0.756	0.604	0.777	0.121	0.048	**0.87**	
DM	0.853	0.593	0.438	0.069	0.344	0.661	0.096	**0.77**

*n = 287. Bolded values on the diagonals of columns 2–5 are the square root values of AVE. AVE, average value extracted; MSV, maximum shared variance; CR, composite reliability; RA, regret aversion; IC, information cascade; RP, risk perception; FL, financial literacy; DM, decision making*.

Hayes's (2013) process macro model 5 was used to test the moderating effect of financial literacy between behavioral biases and investment decisions. Financial literacy was found to have a significant direct effect on the relationship between regret aversion and investment decision (β= −0.270, *p* < 0.048). Therefore, hypothesis 5 is supported.

These interaction terms were plotted +1/−1 SD from the mean of financial literacy. The interaction term between regret aversion and financial literacy is significant and positive. The values of financial literacy against coefficient and significance are (β= −0.003, *p* < 0.000). The results of the regression suggest that it moderates the relationship between regret aversion and investment decisions.

Financial literacy was proposed to have a direct effect on the relationship between information cascade and investment decisions (hypothesis 6). The results show that the interaction between information cascade and investment decision is significant (β = −0.406, *p* < 0.002). These interaction terms were plotted +1/−1 SD from the mean of financial literacy. The interaction term between information cascades and financial literacy is significant and positive. The value of financial literacy is significant (β = −0.024, *p* < 0.000). The results of the regression suggest that financial literacy moderates the relationship between information cascade and investment decisions (Table 9).

**Table 9:** Summary of hypotheses.

**Hypotheses**	**Relationship/Effect**	**Accepted/Rejected**
H1	RA → DM	Accepted
H2	IC → DM	Accepted
H3	RA → RP → DM	Accepted
H4	IC → RP → DM	Accepted
	FL ↓	
H5	RA → DM	Accepted
	FL ↓	
H6	IC → DM	Accepted

The results also reveal the effect of the interaction term between regret aversion and investment decision (β= −0.198, *p* < 0.039). This result suggests that financial literacy strengthens the positive relationship between regret and investment decisions. Thus, hypothesis 5 is supported. The effect of the interaction term between information cascade and investment decision is also significant (β= −0.191, *p* < 0.034). This result suggests that financial literacy strengthens the positive relationship between information cascade and investment decisions. Thus, hypothesis 6 is also supported.

Hayes (2013) also reported a method for evaluating moderation and mediation by an index value, BootLLCI, and BootULCI. The index value did not fall within the upper or lower limit of the class interval. This indicates that the model is perfectly mediation moderation (Table 10) according to the SEM results in AMOS and process macro.

**Table 10:** Index of mediation moderation.

	**Index**	**Boot SE**	**LL (95% CI)**	**UP (95% CI)**
	0.04	0.02	0.004	0.11

*N = 287, B = unstandardized regression coefficient. Bootstrap sample size = 5,000. Confidence interval = 95%. LL, lower limit; UL, upper limit; SE, standard error*.

## Discussion

Since its inception in the 1980s, the concept of behavioral finance has grown within the field of finance. It is based on two approaches: limit to arbitrage and cognitive psychology (Thaler, 1985). According to the limit to arbitrage, investors might not make profits from their investments due to their irrational behavior or limited market knowledge. Meanwhile, cognitive psychology focuses on how individuals think about their decision-making techniques and how risky they feel investments when investing in security markets. Recent behavioral finance studies have uncovered the behavioral biases that influence decisions of investors in specific situations.

The study determined the effects of cognitive biases on investment decisions while considering financial literacy as a moderator in the real estate sector. This study confirmed that biases negatively influence investment decisions. Moreover, this research confirmed that financial literacy mitigates the negative effects of regret aversion bias and information cascade bias on investment decisions. In other words, financially literate investors can overcome judgment errors when making decisions. Njoroge and Gathungu (2013) stated that financially literate investors tend to make better financial decisions with fewer management errors than their financially illiterate counterparts.

All hypotheses presented in this study were accepted. The first aim of this study was to investigate how regret aversion impacts investment decisions; the regression results show that regret aversion has a significant negative effect on investment decisions. This result supports the study's first hypothesis: “Regret aversion significantly affects investment decisions.” Regret aversion is a negative bias that leads investors to make (typically poor) investment decisions based on their regret related to previous decisions. Shimanoff (1984) cited regret aversion as a common negative emotion.

This study also investigated the effect of information cascades on investment decisions. The process macro under regression analysis indicated that information cascades have a significant and negative effect on investment decisions. This result supports the study's third hypothesis: “Information cascades significantly affect investment decisions.” Information cascade bias refers to investors' lack of knowledge about the property, ignorance of specific information that they might have, and tendency to blindly follow the crowd. Alevy et al. (2007) examined an information cascade in which investors have imperfect knowledge about the real estate sector. It cannot be rational to ignore one's knowledge and make decisions based solely on others' decisions.

Another aim of this study was to investigate the mediating effect of risk perceptions on the relationships between cognitive biases and investment decisions. The results indicated that risk perception partially mediates these relationships. This result supports the hypothesis that “Risk perception mediates the relationship between regret aversion and investment decisions.” It also supports the fourth hypothesis: “Risk perception mediates the relationship between information cascade and investment decisions.”

Moreover, Ishfaq et al. (2020) stated that behavioral biases significantly affect risk perceptions, which, in turn, affect investment decisions. In addition, the behavioral biases examined in this paper indirectly influenced investment choices through their impact on risk perception. In other words, risk perception mediates the relationships between behavioral biases and investment decisions.

The fourth study objective was to investigate the moderating effect of financial literacy on the relationship between regret aversion and investment decisions. The regression analysis under process macro indicated that financial literacy weakens the negative relationship between regret aversion and investment decisions. This result supports the hypothesis predicting a “Moderating role of financial literacy in the relationship between regret aversion and investment decisions.” A financially literate investor will reduce any ambiguity involved in an investment decision. Dimmock et al. (2016) examined that financial literacy improves investors' investment decisions and overcomes ambiguity. Thus, financially literate investors can make better financial decisions than inexperienced investors.

Finally, this research investigated the moderating effect of financial literacy in the relationship between information cascade and investment decisions. The results of the process macro regression analysis indicated that financial literacy weakens the negative relationship between information cascade and investment decisions. This result supports the study's sixth hypothesis related to the “Moderating role of financial literacy in the relationship between information cascade and investment decisions.” Investors who rely on professional advice and consult with others tend to make profitable investments because they constantly learn from their advisers (Bachmann and Hens, 2015). Financially literate investors consider expert advice on their financial matters, which helps them profit from their investments.

## Conclusion and Implication

This research examined the direct and indirect mediation effects of risk perception on the relationships between cognitive biases (namely, regret aversion and information cascade) and investment decisions. Moreover, risk perception partially mediates the relationships between cognitive and investment decisions. This research also explored the moderation effect of financial literacy in the relationships of regret aversion information cascade with investment decisions. In this study, correlation and regression were utilized to examine the relationships between variables. Behavioral biases were found to negatively affect investment decisions.

Investors do not always act rationally due to different biases. According to Kumar and Goyal (2016), various inconsistencies in the behaviors of investors distract them from rational and logical decisions and violate standard financial theory. These inconsistencies (referred to as biases or cognitive errors) affect investment decisions.

Risk perception significantly mediates the relationship between behavioral biases and investment decisions. As Ishfaq et al. (2017) stated, behavioral biases significantly affect risk perceptions and investment decisions. Behavioral aspects are essential in the financial market because they affect the financial decisions of investors. Moreover, financial literacy moderates the relationships of regret aversion and information cascade with investment decisions by weakening these negative relationships. According to Dimmock et al. (2016), knowledge of financial literacy improves the financial decisions of investors by reducing the ambiguity involved in such decisions. Decisions of an investor are unlikely to be biased if the investor is financially literate in a specific area, such as the real estate sector of a developing country. An investor should have the financial education required to remove biases from their investment decisions. In addition, the government should encourage investors to become financially literate to protect the real estate sector.

This present study proposed that behavioral biases negatively affect investment decisions of investors, both directly and indirectly, considering risk perception as a mediator and financial literacy as a moderator. Kumar and Goyal (2016) explained that researchers have discovered various inconsistencies in the behavior of investors that distract them from rational and logical decisions and violate standard financial theory. These inconsistencies manifest as behavioral biases or cognitive errors that affect investment decisions. Moreover, Jackson and Orr (2019) revealed that uncertainty in the investment decision-making process could cause asset prices to deviate from their market value and make risky behavior seem irrational.

In this study, we combine the theoretical fields of cognitive psychology and perception of risk along with financial literacy and investment decision literature. Thus, the study makes a theoretical contribution by providing further insights into the cognitive biases and decision-making relationship by exploring how decisions and performance of an individual investor are affected by regret aversion, information cascade, risk perception, and financial literacy. This study provides several practical implications for the government and real estate investors. For instance, the government should conduct seminars and workshops on financial securities knowledge and behavior for real estate investors to reduce biases in their investment decisions, thereby helping the real estate market prosper. Others propose that many investors invest in the real estate sector despite not having formed their own opinions about investing—instead, they blindly follow others. Therefore, investors should attend courses that teach them about the behavioral biases that influence investment decisions, thus enabling them to manage their financing in the real estate sector more effectively.

The results of the study suggested that the investment strategy relying on fast and frugal rules would not result in better returns to investors. Based on our findings, researchers would like to suggest that investors should not rely on cognitive thinking while making investments, but conduct a proper analysis of investment opportunities, develop quantitative investment criteria, and establish investment objectives and constraints, base decisions on their financial capability and experience levels (financial literacy) instead of making investment decisions by using cognitive biases.

Shefrin (2000) stated that practitioners should study behavioral finance to recognize, understand, and learn to avoid their own mistakes and the mistakes of others. The present research proposed that financial literacy can help investors avoid biases, as financially literate investors can easily overcome their biases. This study also provides practical implications to the government related to the fact that many other biases in the real estate sector can influence the decisions of investors. Therefore, is it better to educate investors with education about these behavioral biases so they can avoid decisions that lead to substantial financial losses.

This research has some limitations. The main limitation is related to the short study period. The behavior of an investor is influenced by political and economic conditions in addition to cognitive biases. However, the short study period did not permit us to analyze the effects of such conditions. Second, due to COVID-19, this study was conducted in metropolitan areas of developing economies. Third, this study was conducted using survey questionnaires, which are associated with several general problems. For example, respondents may be hesitant and provide biased responses, which could affect the results.

The present study proposes several recommendations for future studies. First, this study could be done with time series data and compared with cross-sectional data. Second, as mentioned in the previous paragraph, this research was limited to metropolitan areas in a developing country. Therefore, future studies could be conducted across multiple cities or cities in developed countries. Third, a quantitative research technique was used in the present study. Therefore, a quantitative data collection method was used. In the future, qualitative research methods could obtain responses from investors through interviews to accurately describe the problems of interest. Thus, qualitative research should be conducted in the future. Fourth, other variables could be examined in future research, such as personality traits, social norms, investors' attitudes, and investor types, to improve the current understanding of financial investment decisions. Fifth, similar studies can compare the decisions of investors in the stock exchange, real estate, and commodity market sectors. Finally, similar studies can examine differences between short-term and long-term investment intentions.

## Data Availability Statement

The original contributions presented in the study are included in the article/supplementary material, further inquiries can be directed to the corresponding author/s.

## Ethics Statement

The studies involving human participants were reviewed and approved by Riphah International University Faisalabad. Written informed consent to participate in this study was provided by the participants' legal guardian/next of kin. Written informed consent was obtained from the individual(s), and minor(s)' legal guardian/next of kin, for the publication of any potentially identifiable images or data included in this article.

## Author Contributions

MI identifies the problem area and build a hypothesis and do working on the significance of study. MK contributes on the result and discussion chapter. All authors contributed to the article and approved the submitted version.

## Conflict of Interest

The authors declare that the research was conducted in the absence of any commercial or financial relationships that could be construed as a potential conflict of interest.

## Publisher's Note

All claims expressed in this article are solely those of the authors and do not necessarily represent those of their affiliated organizations, or those of the publisher, the editors and the reviewers. Any product that may be evaluated in this article, or claim that may be made by its manufacturer, is not guaranteed or endorsed by the publisher.

## References

[B1] Abrahamson E. (1991). Managerial fads and fashions: the diffusion and rejection of innovations. Acad. Manage. Rev. 16, 586–612.

[B2] Agnew J. R. Szykman L. R. (2005). Asset allocation and information overload: the influence of information display, asset choice, and investor experience. J. Behav. Finan. 6, 57–70. 10.1207/s15427579jpfm0602_2

[B3] Ahmad M. (2020), Does underconfidence matter in short-term and long-term investment decisions? Evidence from an emerging market. Manage. Decis. 59, 172–195. 10.1108/MD-07-2019-0972

[B4] Ahmad M. Shah S. Z. A. (2020). Overconfidence heuristic-driven bias in investment decision-making and performance: mediating effects of risk perception and moderating effects of financial literacy. J. Econ. Admin. Sci. 36. 178–209. 10.1108/JEAS-07-2020-0116

[B5] Alam M. N. Masroor I. Nabi M. N. U. (2020), Does entrepreneurs' risk perception influence firm's rapidity in foreign market entry through moderation of entrepreneurial decision-making approach? Rev. Int. Bus. Strategy 30, 225–243. 10.1108/RIBS-07-2019-0103

[B6] Alba J. W. Hutchinson J. W. (1987). Dimensions of consumer expertise. J. Consum. Res. 12, 411–454. 10.1086/209080

[B7] Alevy J. E. Haigh M. S. List J. A. (2007). Information cascades: evidence from a field experiment with financial market professionals. J. Finan. 62, 151–180. 10.1111/j.1540-6261.2007.01204.x

[B8] Ali Z. Sabir S. Mehreen A. (2019), Predicting engagement performance through firm's internal factors: evidence from textile sector. J. Adv. Manage. Res. 16, 763–780. 10.1108/JAMR-11-2018-0098

[B9] Bachmann K. Hens T. (2015). Investment competence and advice seeking. J. Behav. Exp. Fin. 6, 27–41. 10.1016/j.jbef.2015.03.001

[B10] Bazley W. J. Cronqvist H. Mormann M. (2021). Visual finance: the pervasive effects of red on investor behavior. Manage. Sci. 67, 455–470. 10.1287/mnsc.2020.3747

[B11] Bell D. E. (1982). Regret in decision making under uncertainty. Operat. Res. 30, 961–981. 10.1287/opre.30.5.961

[B12] Bellofatto A. D'Hondt C. De Winne R. (2018). Subjective financial literacy and retail investors' behavior. J. Bank. Finan. 92, 168–181. 10.1016/j.jbankfin.2018.05.004

[B13] Bikhchandani S. Hirshleifer D. Welch I. (1992). A theory of fads, fashion, custom, and cultural change as informational cascades. J. Polit. Econ. 100, 992–1026. 10.1086/261849

[B14] Blanchflower D. G. Oswald A. J. (2004). Well-being over time in Britain and the USA. J. Public Econ. 88, 1359–1386. 10.1016/S0047-2727(02)00168-8

[B15] Bornstein M. H. Jager J. Putnick D. L. (2017). Sampling in development science, situation, shortcoming, solutions and standards. Dev. Rev. 33, 357–370. 10.1016/j.dr.2013.08.003 25580049 PMC4286359

[B16] Brüggen E. C. Hogreve J. Holmlund M. Kabadayi S. Löfgren M. (2017). Financial well-being: a conceptualization and research agenda. J. Bus. Res. 79, 228–237. 10.1016/j.jbusres.2017.03.01332754095

[B17] Bryman A. Bell E. (2011). Business Research Methods, 3rd Edn. Cambridge; New York, NY: Oxford University Press.

[B18] Brzezicka J. Wisniewski R. Figurska M. (2018). Disequilibrium in the real estate market: Evidence from Poland. Land Use Policy 78, 515–531. 10.1016/j.landusepol.2018.06.013

[B19] Çelen B. Kariv S. (2004). Distinguishing informational cascades from herd behavior in the laboratory. Amer. Econ. Rev. 94, 484–498. 10.1257/0002828041464461

[B20] Chen W. Goh M. Zou Y. (2018). Logistics provider selection for omni-channel environment with fuzzy axiomatic design and extended regret theory. Appl. Soft Comput. 71, 353–363. 10.1016/j.asoc.2018.07.019

[B21] Daskalaki C. Kostakis A. Skiadopoulos G. (2014). Are there common factors in individual commodity futures returns? J. Bank. Finan. 40, 346–363. 10.1016/j.jbankfin.2013.11.034

[B22] Daskalaki C. Skiadopoulos G. (2016). The effects of margin changes on commodity futures markets. J. Finan. Stabil. 22, 129–152. 10.1016/j.jfs.2016.01.002

[B23] Dimmock G. S. Kouwenberg R. Mitchell S.O Peijnenburg K. (2016). Ambiguity aversion and household portfolio choice puzzles: empirical evidence. J. Finan. Econ. 119, 559–577. 10.1016/j.jfineco.2016.01.003 28458446 PMC5408951

[B24] Djalilov A. Ülkü N. (2021). Individual investors' trading behavior in Moscow Exchange and the COVID-19 crisis. J. Behav. Exp. Fin. 31, 100549. 10.1016/j.jbef.2021.100549 34545324 PMC8444951

[B25] Duan W. Gu B. Whinston A. B. (2009). Informational cascades and software adoption on the internet: an empirical investigation. MIS Quar. 33, 23–48. 10.2307/20650277

[B26] Fishburn P. C. (1982). Nontransitive measurable utility. J. Mathemat. Psychol. 26, 31–67. 10.1016/0022-2496(82)90034-7

[B27] Fornell C. Larcker D. F. (1981). Structural equation models with unobservable variables and measurement error: algebra and statistics. J. Market. Res. 18. 382–388. 10.1177/002224378101800313

[B28] Frehen R. G. P. Hoevenaars R. P. M. M. Palm F. C. Schotman P. C. (2008). Regret aversion and annuity risk in defined contribution pension plans. Insurance 42, 1050–1106. 10.1016/j.insmatheco.2008.01.001

[B29] George D. (2011). SPSS for Windows Step By Step: A Simple Study Guide and Reference, 17.0 Update. Boston, MA: Pearson Education India.

[B30] Ghasemi A. Saleh Z. (2012). Normality tests for statistical analysis: a guide for non-statisticians. Int. J. Endocrinol. Metab. 10, 486. 10.5812/ijem.3505 23843808 PMC3693611

[B31] Giesler M. Veresiu E. (2014). Creating the responsible consumer: moralistic governance regimes and consumer subjectivity. J. Consum. Res. 41, 840–857. 10.1086/677842

[B32] Gitau G. G. Kiragu D. N. U. Riro G. K. (2019). Effect of heuristic factors and real estate investment in Embu County, Kenya. Int. J. Acad. Res. Account. Finan. Manage. Sci. 8, 30–38. 10.6007/IJARAFMS/v8-i4/5183

[B33] Hair J. F. Anderson R. E. Tatham R. L. William C. (1998) Multivariate Data Analysis. Upper Saddle River, NJ: Pearson Prentice Hall.

[B34] Hair J. F. Black W. C. Babin B. J. Anderson R. E. (2010). Multivariate Data Analysis: A Global Perspective, 7th Edn. Upper Saddle River, NJ: Pearson Prentice Hall.

[B35] Hair J. F. Black W. C. Babin B. J. Anderson R. E. Tatham R. L. (2006). Multivariate Data Analysis, Vol. 6. Upper Saddle River, NJ: Pearson Prentice Hall.

[B36] Hassan Al-Tamimi H. A. Anood Bin Kalli A. (2009). Financial literacy and investment decisions of UAE investors. J. Risk Finan. 10, 500–516. 10.1108/15265940911001402

[B37] Hayes A. F.. (2013). Introduction to Mediation, Moderation, and Conditional Process Analysis: A Regression-Based Approach. New York, NY: The Guilford Press. J. Educ. Meas. 51, 335–337. 10.1111/jedm.12050

[B38] Hilgert M. A. Hogarth J. M. (2002). Financial knowledge, experience and learning preferences: preliminary results from a new survey on financial literacy. Consum. Interest Annu. 48, 1–7.

[B39] Houghton S. M. Simon M. Aquino K. Goldberg C. B. (2000). No safety in numbers. Group Organ. Manage. 25, 325–353. 10.1177/1059601100254002

[B40] Huber R. E. Klucharev V. Rieskamp J. (2014). Neural correlates of informational cascades: brain mechanisms of social influence on belief updating. Soc. Cogn. Affect. Neurosci. 10, 589–597. 10.1093/scan/nsu090 24974396 PMC4381243

[B41] Huston S. J. (2010). Measuring financial literacy. J. Consum. Aff. 44, 296–316. 10.1111/j.1745-6606.2010.01170.x

[B42] Idris F. H. Krishnan K. S. D. Azmi N. (2013). Relationship between financial literacy and financial distress among youths in Malaysia-an empirical study. Geografia 9, 106–117.

[B43] Idris F. H. Krishnan K. S. D. Azmi N. (2017). Relationship between financial literacy and financial distress among youths in Malaysia-an empirical study. Geografia Malay. J. Soc. Space 9, 106–117.

[B44] Ishfaq M. Maqbool Z. Akram S. Tariq S. Khurshid M. K. (2017). Mediating role of risk perception between cognitive biases and risky investment decision: empirical evidence from Pakistan's equity market. J. Manage. Sci. 11, 1–9.

[B45] Ishfaq M. Nazir M. S. Qamar M. A. J. Usman M. (2020). cognitive bias and the extraversion personality shaping the behavior of investors. Front. Psychol. 11:2513. 10.3389/fpsyg.2020.556506 33178066 PMC7593711

[B46] Isidore R. R. Christie P. (2019), The relationship between the income behavioural biases. J. Econ. Finan. Admin. Sci. 24, %127–144. 10.1108/JEFAS-10-2018-0111

[B47] Jackson C. Orr A. (2019). Investment decision-making under economic policy uncertainty. J. Proper. Res. 36, 153–185. 10.1080/09599916.2019.1590454

[B48] Jariwala V. H. (2015). Analysis of financial literacy level of retail individual investors of Gujarat state and its effect on investment decision. J. Bus. Finan. Librar. 20, 133–158. 10.1080/08963568.2015.977727

[B49] Jiang J. Liao L. Wang Z. Xiang H. (2019). Financial literacy and retail investors' financial welfare: Evidence from mutual fund investment outcomes in China. Pacific Basin Finan. J. 59, 101242. 10.1016/j.pacfin.2019.101242

[B50] Kahan M. Klausner M. (1996). Path dependence in corporate contracting: increasing returns, herd behavior and cognitive biases. Washing. Univ. Law Rev. 74, 347.

[B51] Kahneman D. Tversky A. (1979). Prospect theory: an analysis of decision under risk. Econometrica 47, 263–291.

[B52] Kaur S. J. Bharucha J. (2021). The emerging mutual fund industry in India: an impact analysis of investors' awareness on investment behaviour. Int. J. Bus. Globalis. 27, 51–69. 10.1504/IJBG.2021.111960

[B53] Khan M. T. I. Tan S.-H. Gan G. G. G. (2018). Advanced financial literacy of Malaysian Gen Y investors and its consequences. Margin J. Appl. Econ. Res. 13, 83–108. 10.1177/0973801018800085

[B54] Kim J. (2001). Financial knowledge and subjective and objective financial well-being. Consum. Inter. Annu. 47, 1–3.

[B55] Kim J. Lee H.-H. (2008). Consumer product search and purchase behaviour using various retail channels: the role of perceived retail usefulness. Int. J. Consum. Stud. 32, 619–627. 10.1111/j.1470-6431.2008.00689.x

[B56] Kline R. B. (1998). Principles and Practice of Structural Equation Modeling. New York, NY: The Guilford Press.

[B57] Knight F. H. (1921). Risk, Uncertainty and Profit. University of Illinois at Urbana-Champaign's Academy for Entrepreneurial Leadership Historical Research in Entrepreneurship.

[B58] Kumar S. Goyal N. (2016). Evidence on rationality and behavioural biases in investment decision making. Qual. Res. Finan. Mark. 8, 270–287. 10.1108/QRFM-05-2016-0016

[B59] Larrick R. P. Boles T. L. (1995). Avoiding regret in decisions with feedback: a negotiation example. Organ. Behav. Hum. Decis. Process. 63, 87–97. 10.1006/obhd.1995.1064

[B60] Levy D. S. Frethey-Bentham C. Cheung W. K. S. (2020). Asymmetric framing effects and market familiarity: experimental evidence from the real estate market. J. Proper. Res. 37, 85–104. 10.1080/09599916.2020.1713858

[B61] Lones S. Peter S. (2000). Pathological outcomes of observational learning. Econ. Econ. Soc. 68, 371–398. 10.1111/1468-0262.00113

[B62] Loomes G. Sugden R. (1982). Regret theory: an alternative theory of rational choice under uncertainty. Econ. J. 92, 805–824. 10.2307/2232669

[B63] Lusardi A. Mitchell O. S. (2011). Financial literacy around the world: an overview. J. Pens. Econ. Finan. 10, 497–508. 10.1017/S1474747211000448 28553190 PMC5445931

[B64] Lusardi A. Mitchell O. S. (2014). The economic importance of financial literacy: theory and evidence. J. Econ. Literat. 52, 5–44. 10.1257/jel.52.1.5 28579637 PMC5450829

[B65] Mellers B. A. (2000). Choice and the relative pleasure of consequences. Psychol. Bull. 126, 910–924. 10.1037/0033-2909.126.6.910 11107882

[B66] Naseem S. Mohsin M. Hui W. Liyan G. Penglai K. (2021). The investor psychology and stock market behavior during the initial era of COVID-19: a study of China, Japan, and the United States. Front. Psychol. 12:626934. 10.3389/fpsyg.2021.626934 33643158 PMC7902781

[B67] Nguyen L. T. M. Gallery G. Newton C. (2016). The influence of financial risk tolerance on investment decision-making in a financial advice context. Aust. Account. Bus. Finan. J. 10, 3–22. 10.14453/aabfj.v10i3.2

[B68] Nguyen T. A. N. Rozsa Z. (2019). Financial literacy and financial advice seeking for retirement investment choice. J. Competit. 11, 70–83. 10.7441/joc.2019.01.05

[B69] Njoroge C. W. Gathungu J. M. (2013). The effect of entrepreneurial education and training on development of small and medium size enterprises in Githunguri District - Kenya International. J. Educ. Res. 1, 1–22.

[B70] Noctor M. Stoney S. Stradling R. (1992), “Financial Literacy”, A Report Prepared for the National Westminster Bank, London.

[B71] Norizan A. R. B. Pee A. N. C. (2021). Using activity theory to review internet technology engagement by real estate negotiator in Malaysia towards agency best practice. J. Environ. Treat. Techniq. 9, 72–76. 10.47277/jett/9(1)76

[B72] Nunnally J. C. (1978), Psychometric Theory. New York, NY: McGraw-Hill

[B73] Odean T. (1998a). Are investors reluctant to realize their losses? J. Finan. 53, 1775–1798. 10.1111/0022-1082.00072

[B74] Odean T. (1998b), Volume, volatility, price and profit when all trades are above average, J. Finan. 53, 1887–1934. 10.1111/0022-1082.00078

[B75] Pandey R. Jessica V. M. (2018). Measuring behavioural biases affecting real estate investment decisions in India: using IRT. Int. J. Hous. Markets Anal. 11, 648–668. 10.1108/IJHMA-12-2017-0103

[B76] Pandey R. Jessica V. M. (2019). Sub-optimal behavioural biases and decision theory in real estate. Int. J. Hous. Markets Anal. 12, 330–348. 10.1108/IJHMA-10-2018-0075

[B77] Pfnur A. Wagner B. (2020). Transformation of the real estate and construction industry: empirical findings from Germany. J. Bus. Econ. 90, 975–1019. 10.1007/s11573-020-00972-4

[B78] Podsakoff P. M. MacKenzie S. B. Lee J. Y. Podsakoff N. P. (2003). Common method biases in behavioral research: a critical review of the literature and recommended remedies. J. Appl. Psychol. 88, 879–903. 10.1037/0021-9010.88.5.879 14516251

[B79] Ramalho T. B. Forte D. (2019). Financial literacy in Brazil–do knowledge and self-confidence relate with behavior? RAUSP Manage. J. 54, 77–95. 10.1108/RAUSP-04-2018-0008

[B80] Rasheed M. H. Rafique A. Zahid T. Akhtar M. W. (2018). Factors influencing investor's decision making in Pakistan. Rev. Behav. Finan. 10, 70–87. 10.1108/RBF-05-2016-0028

[B81] Robinson A. T. Marino L. D. (2013). Overconfidence and risk perceptions: do they really matter for venture creation decisions? Int. Entrepreneur. Manage. J. 11, 149–168. 10.1007/s11365-013-0277-0

[B82] Sahin A. M. Ates S. Demircan L. S. M. (2016). Impact of financial literacy on the behavioral biases of individual stock investors: evidence from Borsa Istanbul. Bus. Econ. Res. J. 7, 1–19. 10.20409/berj.2016321805

[B83] Sartori R. Ceschi A. (2011). Uncertainty and its perception: experimental study of the numeric expression of uncertainty in two decisional contexts. Qual. Quan. 45, 187–198. 10.1007/s11135-010-9365-1

[B84] Sattar M. A. Toseef M. Sattar M. F. (2020). Behavioral finance biases in investment decision making. Int. J. Account. Finan. Risk Manage. 5, 69–75. 10.11648/j.ijafrm.20200502.11

[B85] Savage L. J. (1951). The theory of statistical decision. J. Amer. Stat. Assoc. 46, 55–67. 10.1080/01621459.1951.10500768

[B86] Scott S. G. Bruce R. A. (1995), Decision-making style: the development assessment of a new measure. Educ. Psychol. Measure. 55, 818–831. 10.1177/001316449505500501715324871

[B87] Seiler M. J. (2012). Forward and falsely induced reverse information cascades. J. Behav. Finan. 13, 226–240. 10.1080/15427560.2012.708688

[B88] Shefrin H. (2000). Beyond Fear and Greed: Understanding Behavioral Finance and the Psychology of *Investing*. Harvard Business School Press.

[B89] Shiller R. J. (2003). From efficient markets theory to behavioral finance. J. Econ. Perspect. 17, 83–104. 10.1257/089533003321164967

[B90] Shimanoff S. B. (1984). Commonly named emotions in everyday conversations. Percept. Motor Skills 58, 514. 10.2466/pms.1984.58.2.514

[B91] Sindhu A. Ramsay L. Sanderson L.-A. Stonehouse R. Li R. Condie J.. (2014). Gene-based SNP discovery and genetic mapping in pea. Theoret. Appl. Genet. 127, 2225–2241. 10.1007/s00122-014-2375-y 25119872 PMC4180032

[B92] Singh R. Bhowal A. (2010). Risk perception of employees with respect to equity shares. J. Behav. Finan. 11, 177–183. 10.1080/15427560.2010.507428

[B93] Sitkin S. B. Weingart L. R. (1995). Determinants of risky decision-making behavior: a test of the mediating role of risk perceptions and propensity. Acad. Manage. J. 38, 1573–1592. 10.5465/256844

[B94] Slovic P. (1987). Perception of risk. Science 236, 280–285. 3563507 10.1126/science.3563507

[B95] Spoerri A. (2008). Authority and ranking effects in data fusion. J. Amer. Soc. Inform. Sci. Technol. 59, 450–460. 10.1002/asi.20760

[B96] Statman M. (2014). Behavioural finance: finance with normal people. Borsa Istanb. Rev. 14,65–73. 10.1016/j.bir.2014.03.001

[B97] Thaler R. (1980). Toward a positive theory of consumer choice. J. Econ. Behav. Organ. 1, 39–60. 10.1016/0167-2681(80)90051-7

[B98] Thaler R. H. (1985). Mental accounting and consumer choice. Market. Sci. 4,199–214. 10.1287/mksc.4.3.199

[B99] Tsiros M. Mittal V. (2000). Regret: a model of its antecedents and consequences in consumer decision making. J. Consum. Res. 26, 401–417. 10.1086/209571

[B100] Wang Q. Yang X. Xi W. (2018). Effects of group arguments on rumor belief and transmission in online communities: an information cascade and group polarization perspective. Inform. Manage. 55, 441–449. 10.1016/j.im.2017.10.004

[B101] Weber E. U. (2004). Perception matters: psychophysics for economists, in The Psychology and Economic Decisions, Vol. 2, eds Carrillo J. Brocas I. (Oxford: Oxford University Press), 163–176.

[B102] Weber E. U. Blais A. R. Betz N. E. (2002). A domain-specific risk-attitude scale: measuring risk perceptions and risk behaviors. J. Behav. Decis. Mak. 15, 263–290. 10.1002/bdm.414

[B103] Weber M. Camerer C. F. (1998). The disposition effect in securities trading: an experimental analysis. J. Econ. Behav. Organ. 33, 167–184. 10.1016/S0167-2681(97)00089-9

[B104] Yang Y. Wang J. (2018). SMAA-based model for decision aiding using regret theory in discrete Z-number context. Appl. Soft Comput. 65, 590–602. 10.1016/j.asoc.2018.02.001

[B105] Zahera S. A. Bansal R. (2018). Do investors exhibit behavioral biases in investment decision making? A systematic review. Qual. Res. Finan. Mark. 10, 210–251. 10.1108/QRFM-04-2017-0028

[B106] Zeelenberg M. (1999). Anticipated regret, expected feedback and behavioral decision making. J. Behav. Decis. Mak. 12, 93–106.

[B107] Zeelenberg M. Beattie J. Pligt J. de van der Vries K. (1996). Consequences of regret aversion: effects of expected feedback on risky decision making. Organ. Behav. Hum. Decis. Process. 65, 148–158. 10.1006/obhd.1996.0013

[B108] Zeelenberg M. Pieters R. (2004). Consequences of regret aversion. Organ. Behav. Hum. Decis. Process. 93, 155–168. 10.1016/j.obhdp.2003.10.001

[B109] Zhuo J. Li X. Yu C. (2021). Parameter behavioral finance model of investor groups based on statistical approaches. Quar. Rev. Econ. Finan. 80, 74–79. 10.1016/j.qref.2021.01.012

